# Evaluating an Artificial Intelligence software for opportunistic low bone mineral density and osteoporosis screening: a validation study

**DOI:** 10.1093/jbmrpl/ziaf191

**Published:** 2025-12-15

**Authors:** Angela M Auriat, Milin Patel, Liying Zhang, Victoria Li, Angela Kwan, Eugene Leung, Richard I Aviv

**Affiliations:** Department of Radiology, Radiation Oncology and Medical Physics, Faculty of Medicine, University of Ottawa, Ottawa, ON K1H 8L6, Canada; The Ottawa Hospital Research Institute, Ottawa, ON K1H 8L6, Canada; Brain and Mind Institute, University of Ottawa, Ottawa, ON K1H 8L6, Canada; Department of Cellular and Molecular Medicine, University of Ottawa, Ottawa, ON KlH 8L6, Canada; Department of Radiology, Radiation Oncology and Medical Physics, Faculty of Medicine, University of Ottawa, Ottawa, ON K1H 8L6, Canada; The Ottawa Hospital Research Institute, Ottawa, ON K1H 8L6, Canada; The Ottawa Hospital Research Institute, Ottawa, ON K1H 8L6, Canada; Department of Radiology, Radiation Oncology and Medical Physics, Faculty of Medicine, University of Ottawa, Ottawa, ON K1H 8L6, Canada; The Ottawa Hospital Research Institute, Ottawa, ON K1H 8L6, Canada; Division of Nuclear Medicine and Molecular Imaging, Department of Medicine, The Ottawa Hospital, Ottawa, ON K1H 8L6, Canada; Nuclear Medicine & Molecular Imaging, Faculty of Medicine, University of Ottawa, Ottawa, ON K1H 8L6; Department of Radiology, Radiation Oncology and Medical Physics, Faculty of Medicine, University of Ottawa, Ottawa, ON K1H 8L6, Canada; The Ottawa Hospital Research Institute, Ottawa, ON K1H 8L6, Canada

**Keywords:** osteoporosis, screening, DXA, radiographs, low bone mineral density

## Abstract

Despite the profound prevalence and fracture risk of osteoporosis, access to the gold standard DXA scans remains limited, especially in rural communities. Rho is an artificial intelligence software that can identify individuals at risk of low BMD and osteoporosis using radiographs. This study will independently validate Rho software by evaluating its performance against DXA in a retrospective cohort of patients. We conducted a retrospective study of 4878 patients (mean age 70 ± 10, 80% female), with DXA acquired within 1 yr of a radiograph. The area under the curve (AUC) was calculated to evaluate the performance of Rho in identifying patients at risk for low BMD (T-Score < −1) and osteoporosis (T-Score ≤ −2.5). Further subgroup analyses were performed based on radiograph location, sex, and rural vs urban populations. The overall AUC for predicting low BMD was 0.840 (95% CI: 0.831-0.848), with an optimal Rho score threshold of 6. For osteoporosis prediction, the AUC was 0.815 (95% CI: 0.806-0.824), with an optimal Rho score threshold of 7. Rural and urban populations have strong AUCs for low BMD (AUC = 0.873; 0.873) and osteoporosis (AUC = 0.865; 0.812). Likewise, Rho demonstrated strong, comparable (*p* > .25) performance in both men and women for prediction of low BMD. Although, optimal cutoffs differed between females and males for both low BMD and osteoporosis. Rho demonstrated high effectiveness in identifying patients at risk for low BMD and osteoporosis. The findings support Rho as an opportunistic screening tool and may fill a clinical gap in communities lacking access to DXA.

## Introduction

The detection of low BMD is a key to early diagnosis and management of osteoporosis.^1^ In the USA, more than 10 million individuals are living with osteoporosis; it is estimated that at least 1 in 3 women and 1 in 5 men will suffer from an osteoporotic fracture during their lifetime.^2^ Due in part to the loss of BMD, the risk of fractures increases with age, posing a significant cost to the healthcare system—exceeding $19 billion annually in the USA.^2^ Increased mortality and morbidity are associated with osteoporosis pathological fractures.^3^^,^^4^ Current recommendations on testing for osteoporosis include a measure of BMD utilizing DXA. This gold standard technique involves the use of polychromatic X-ray spectra at different energies to infer bone density.^5^ However, less than 20% of patients with osteoporosis-related fractures undergo a diagnostic test or receive adequate treatment for osteoporosis, highlighting a significant gap in healthcare delivery.^2^

The issue of underdiagnosis and treatment for osteoporosis is exacerbated in marginalized populations, specifically racial and ethnic minority groups, and those of lower socioeconomic status. Significant disparities in DXA screening in racial and ethnic minority groups are reported^6^^,^^7^ with higher rates of unmanaged osteoporosis, for example, in the Métis population, and other lower socioeconomic groups.^8^^,^^9^ Métis are a group of distinct indigenous people in Canada, with European and indigenous ancestry.^8^ There are also significantly lower rates of BMD testing among the Métis population.^8^ Lack of early detection and screening results in greater fracture rates among these groups.^10^ Many patients lack access to appropriate screening indicating a greater need for more accessible and opportunistic screening methods that can be integrated into routine practice.

While DXA scans are considered as the clinical gold standard for measuring BMD, their implementation is limited due to the need for specialized equipment, training, and knowledge to interpret the results accurately.^11^ These requirements contribute to the higher costs and limited accessibility of DXA scans, thus reducing access to screening. In comparison, radiographs are a part of routine clinical practice and are widely available including in marginalized communities. Rho is a Health Canada and FDA authorized Software-as-a-Medical-Device that can interface with standard digital radiographs to identify patients at increased risk of low bone density.^12^ This technology can increase screening at a population level and specifically in communities challenged by DXA access. Rho utilizes an artificial intelligence (AI)-trained model to analyze frontal radiographs of the LS, thoracic spine, chest, hand/wrist, pelvis, or knee. This model was developed by 16Bit and trained using a single machine-learning algorithm from a dataset with no overlap to the one assessed in this manuscript.^10^ Based on the features of the bone and soft tissue, Rho outputs an integer score from 1 to 10, where 1 is least likely to have low BMD and 10 most likely.

Our institution serves a diverse population including marginalized communities in rural settings identifiable through a postal code filter. Currently, there is only one study that reports the performance of Rho in three urban datasets.^10^ The external validation of Rho is important as the integration of AI technology into standard clinical practice can reduce the healthcare burden of osteoporosis by facilitating targeted screening, specifically in underserved populations. The goal of this study is to independently validate Rho software in a broad patient population by evaluating its performance against DXA gold standard in a retrospective cohort of patients served by our institution.

## Materials and methods

### Study population

**Figure 1:**
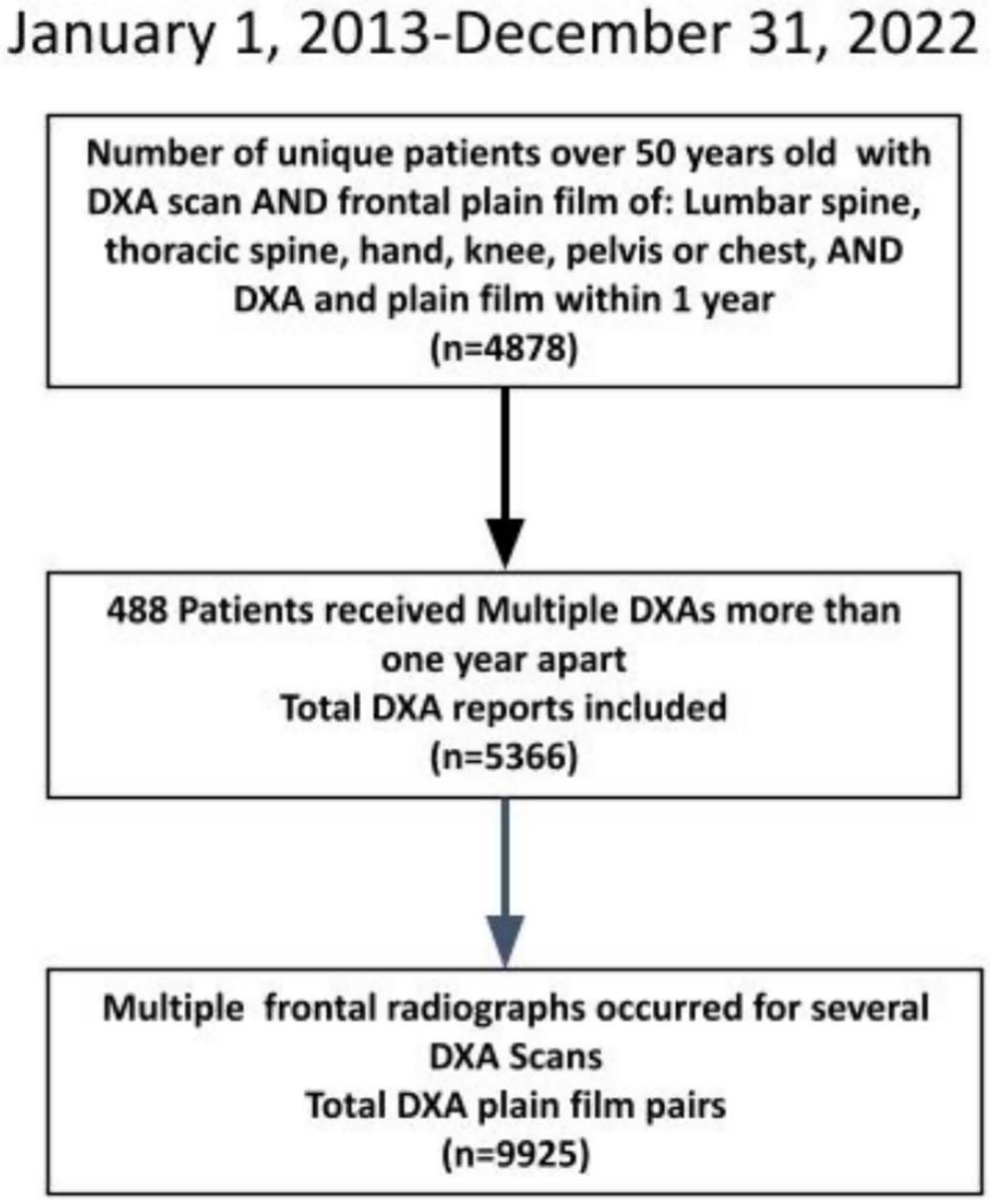
Flowchart of patient selection.

The study was approved by the local University Research Ethics Board (REB). Due to the retrospective nature of this study, our REB waived informed consent. Participants were selected based on the age and radiograph requirements of the Rho program from the rural and urban population served by our institution. Study cohort was identified by searching all hospital electronic medical record for qualifying cases occurring between January 1, 2013 and December 31, 2022. We identified cases that met all 4 search criteria: (1) individuals over 50 yr old; (2) DXA scan; (3) frontal radiograph of any of the following: lumbar spine, thoracic spine, hand, knee, pelvis, or chest; and (4) 2 and 3 within 365 d of each other. About 4878 retrospective patients met these criteria, undergoing a DXA within 365 d prior to or following the radiograph (mean absolute difference, SD; 116.5, 98.9). Several of these patents had multiple DXA scans, and multiple radiographs, resulting in a total of 5366 DXA scans and 9925 radiographs (Figure 1). We did not exclude any radiographs based on clinical features such as hardware, fracture, or infection, allowing for a sample that is representative of a broad and typical radiographic patient population.

**Figure 2:**
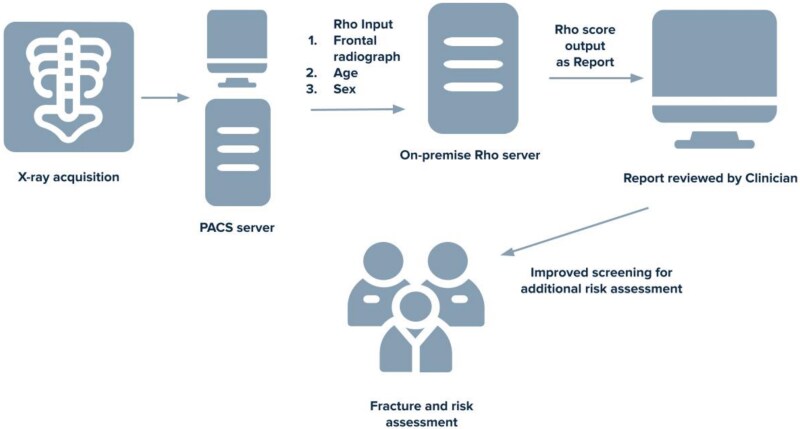
Demonstrating the workflow of Rho software from X-ray acquisition to the clinic.

DXA and radiographs were obtained from multiple machines with techniques required by our institution. The images along with patient age and sex were entered into Rho (Figure 2). Rho outputs a Rho score, rather than a T-score, as it is not intended to diagnose or rule out disease. The Rho score ranges from 1 to 10 and correlates with a patient’s likelihood of having low BMD (−2.5 < T-score < −1) or osteoporosis (T-score ≤ −2.5). Specific details of the Rho algorithm and radiographic requirements have been previously reported by the developer.^10^ Presumably, the algorithm is detecting subtle features in the image related to reduced bone quality (eg, microarchitecture) associated with osteoporosis. Patient demographics, such as sex, and age, DXA results, and geographic location data were extracted from electronic medical records. The distinction between rural and urban communities was determined using patient postal codes. These codes designated by Canada Post identify rural and urban delivery areas.^13^

### Diagnosis of osteoporosis and low BMD

We utilized the International Society for Clinical Densitometry recommendation to reference female peak BMD in the T-score calculation.^14^ All participants had 1-3 DXA scans of FN (5133 scans), spine (4544 scans), or TH (4928 scans). In the case of multiple DXA locations classification of normal bone density (T-Score ≥ −1), low BMD (−2.5 < T-Score < −1) or osteoporosis (T-Score ≤ −2.5), was based on the lowest T-score as recommended by the Canadian Association of Radiologists.^2^

### Statistical analyses

The performance of Rho was assessed using the area under the receiver operating characteristic curve (AUC). To demonstrate a clinically meaningful performance of Rho to identify patients at risk of low BMD or osteoporosis, an AUC lower 95% CI threshold limit of >0.7 was set, as it is statistically accepted as an acceptable discrimination level.^15^ To evaluate the performance of the Rho software in predicting low BMD or osteoporosis, we calculated the AUC overall as well as stratified by radiograph locations, sex, and geographic location (rural or urban). The optimal Rho score thresholds were determined using Youden’s index method. Bootstrap resampling (*n* = 2000) was employed to estimate 95% CIs for both AUC and the optimal Rho score thresholds. Optimal Rho score thresholds will be presented with a 95% CI; however, these scores can’t be directly compared. Presentation of the optimal Rho score threshold will be provided as a guide to direct clinical interpretation of the Rho in varying populations. To determine statistically significant differences in AUC values, a chi-square with one degree of freedom was employed. A *p*-value less than .05 was considered statistically significant from the ꭓ^2^ distribution.

When multiple X-ray scans were associated with a single DXA exam, we calculated the coefficient of variation (CV) for all Rho scores to determine the relative dispersion of data points (Rho scores) for each DXA scan.

Confusion matrixes will be generated with the optimal Rho cut points for classifying low BMD and osteoporosis, demonstrating the number of cases classified as true positive (TP), true negative (TN), false positive (FP), and false negative (FN). From this table, we will calculate the sensitivity (SE; True Positive = TP/(TP + FN)), specificity (SP; True Negative = TN/(TN + FP)), positive predictive value (PPV; PPV = TP/(TP + FP)), and negative predictive value (NPV; NPV = TN/(TN + FN)), along with their 95% CIs.

## Results

### Dataset

The study included a dataset of 9925 frontal radiographs from a total of 4878 patients with demographic data, radiograph location, distribution of patients with normal BMD, low BMD, and osteoporosis, and Rho scores indicated in Table 1.

**Table 1:** Demographic characteristics of patients.

	Participants (*n* = 4853)
**Sex** **Female**	3862 (79.6%)
**Age, yr** **Mean (SD)**	70.5 (10.1)
**Female** **Male**	69.3 (10.0) 71.9 (9.8)
**Days between X-Ray and DXA**	
**Mean (SD)**	116.5 (98.9))
**Radiograph location**	
**Chest PA**	1718 (35%)
**Hand-wrist AP**	1437 (30%)
**Knee**	2500 (51%)
**Lumbar**	1135 (23%)
**Pelvis**	2649 (55%)
**Thoracic**	486 (10%)
**DXA scan**	
**Normal**	1369 (28%)
**Low BMD**	2717 (56%)
**Osteoporosis**	1280 (26%)
**Geographic** **Rural**	247 (5.1%)
**Rho score**	
**1**	235 (4.9%)
**2**	242 (5.0%)
**3**	499 (10%)
**4**	847 (17%)
**5**	1397 (29%)
**6**	1935 (40%)
**7**	1944 (40%)
**8**	1434 (30%)
**9**	771 (16%)
**10**	621 (13%)

### Primary effectiveness endpoint analysis

The overall AUC for predicting low BMD for all locations was 0.840 (95% CI: 0.831-0.848), with an optimal Rho threshold of 6 (95% CI: 6-6; Table 2). The lower limit of 95% CI of AUC was 84%, exceeding the 70% threshold. The overall AUC for predicting osteoporosis for all locations was 0.815 (95% CI: 0.806-0.824; Table 2), with an optimal Rho threshold of 7 (95% CI: 7-8).

**Table 2:** AUC and optimal Rho score thresholds for low BMD and osteoporosis in total dataset and by radiograph locations.

Low BMD					
**Analysis group**	**Number of datapoints**		**AUC** **(95% CI)**	**Optimal Rho score cutpoint (95% CI)**	***p*-value**
	**Total**	**Low BMD (%)**			
**Entire study**	9925	7449 (75)	0.840 (0.831-0.848)	6 (6-6)	
**By X-ray locations**					
**Chest**	1718	1326 (77)	0.829 (0.806-0.850)	7 (6-7)	**<.0001**
**Hand**	1437	1118 (78)	0.791 (0.763-0.819)	6 (6-6)	**<.0001**
**Knee**	2500	1819 (73)	0.805 (0.787-0.823)	6 (6-6)	**<.0001**
**Lumbar**	1135	866 (76)	0.858 (0.833-0.882)	6 (6-6)	**.0171**
**Pelvic**	2649	1938 (73)	0.892 (0.879-0.905)	6 (6-6)	Reference
**Thoracic**	486	382 (79)	0.855 (0.820-0.889)	7 (7-8)	.0523
**Osteoporosis**					
**Analysis group**	**Number of datapoints**		**AUC** **(95% CI)**	**Optimal Rho score cutpoint (95% CI)**	***p*-value**
	**Total**	**Osteoporosis (%)**			
**Entire study**	9925	2506 (25)	0.815 (0.806-0.824)	7 (7-8)	
**By X-ray locations**					
**Chest**	1718	499 (29)	0.794 (0.771-0.815)	8 (7-8)	**<.0001**
**Hand**	1437	318 (22)	0.764 (0.734-0.791)	7 (7-8)	**<.0001**
**Knee**	2500	540 (22)	0.785 (0.763-0.805)	7 (7-7)	**<.0001**
**Lumbar**	1135	333 (29)	0.822 (0.796-0.846)	7 (7-7)	**.0002**
**Pelvic**	2649	661 (25)	0.876 (0.862-0.889)	8 (8-8)	Reference
**Thoracic**	486	155 (32)	0.800 (0.759-0.843)	8 (8-9)	**.0005**

Abbreviations: AUC, area under the curve; BMD, bone mineral density; CI confidence Interval.

### Radiograph location-based Rho performance

In both low BMD and osteoporosis screening, all radiographs had high (>70%) AUC across all locations (Table 2). For predicting low BMD, Rho performance on the pelvic radiograph demonstrated an AUC of 0.892 (95% CI: 0.879-0.905), which was significantly better than performance of chest, hand, knee, and lumbar radiographs (*p* ≤ .0171; Table 2). For predicting osteoporosis, Rho performance on pelvic radiographs was 0.876 (95% CI: 0.862-0.889) and was significantly better than performance for chest, hand, knee, lumbar, and thoracic locations (*p* ≤ .0005; Table 2).

### Sex-based Rho performance

The performance of Rho in predicting low BMD and osteoporosis was evaluated separately for males and females. The AUC for prediction of low BMD, in males (AUC 0.848, 95% CI: 0.830-0.865; Table 3), and in females (AUC 0.836, 95% CI: 0.827-0.847; Table 3) was not significantly different (*p* = .26). Similarly for osteoporosis screening, the AUC for males 0.833 (95% CI: 0.813-0.852) and for females 0.820 (95% CI: 0.810-0.830) did not significantly differ (*p* = .25). The optimal Rho score thresholds for low BMD screening was 6 for females and 5 in males. Similarly for the prediction of osteoporosis, the optimal Rho score cutoff was 8 for women and 6 for men.

**Table 3:** Area under the curve and the optimal Rho score thresholds for low BMD and osteoporosis in males, females, urban, and rural.

Low BMD					
**Analysis group**	**Number of datapoints**		**AUC (95% CI)**	**Optimal Rho score cutpoint (95% CI)**	***p*-value**
	**Total**	**Low BMD (%)**			
**Male**	1987	1325 (66)	0.848 (0.830-0.865)	5 (5-5)	.2576
**Female**	7938	6124 (77)	0.836 (0.827-0.847)	6 (6-6)	
**Urban**	9394	7081 (75)	0.837 (0.828-0.846)	6 (6-6)	**.0341**
**Rural**	531	368 (69)	0.873 (0.839-0.902)	6 (5-6)	
**Analysis group**	**Number of datapoints**		**AUC (95% CI)**	**Optimal Rho score cutpoint (95% CI)**	***p*-value**
	**Total**	**Osteoporosis (%)**			
**Osteoporosis**					
**Male**	1987	467 (24)	0.833 (0.813-0.852)	6 (6-7)	.2458
**Female**	7938	2039 (26)	0.820 (0.810-0.830)	8 (8-8)	
**Urban**	9394	2371 (25)	0.812 (0.802-0.821)	8 (7-8)	**.0025**
**Rural**	531	135 (25)	0.865 (0.829-0.897)	7 (7-8)	

Abbreviations: AUC, area under the curve; BMD, bone mineral Density; CI, confidence interval.

### Rho performance in rural populations

The AUC for Rho performance was consistently high (>0.80) in the rural and urban groups. The proportion of patients within each Rho score was found to be similar across both population subgroups with a statistically insignificant −0.51% to −1.19% percent difference for each Rho score category (*p* > .05). For predicting low BMD, in the urban group, the AUC was 0.837 (95% CI: 0.828-0.846) and differed significantly from the rural group AUC which was 0.873 (95% CI: 0.839-0.902, *p* = .03; Table 3). The optimal Rho score threshold was 6 in both groups. In osteoporosis screening, the AUC for the urban group was 0.812 (95% CI: 0.802-0.821) and differed significantly from the rural group AUC which was 0.865 (95% CI: 0.829-0.897, *p* = .003; Table 3). The optimal Rho score for the prediction of osteoporosis was 8 in the urban group and 7 for the rural group.

### Variation of Rho scores for each DXA

Lower CV indicates less variability and higher stability. The mean CV (95% CI) per DXA scan was 17.0% (16.4%-17.6%), and the median CV (95% CI) was 12.9% (12.8%-14.2%) per DXA scan. The mean and median CV per DXA scan was below the 20% threshold commonly used to demonstrate low variability.^16^

### Confusion matrix

Confusion matrixes were generated with cut points of Rho score 7 and 8. The number of true positive (TP), true negative (TN), false positive (FP), and false negative (FN) osteoporosis and low BMD cases were identified (Table S1). The SE, SP, PPV, and NPV, along with their 95% CI are provided in Table 4.

**Table 4:** Confusion matrix, summarizing performance/validity of Rho scores 7 and 8.

Confusion matrix for low BMD—Rho score of 7					Confusion matrix for low BMD—Rho score of 8			
	SE % (95% CI)	SP% (95% CI)	PPV% (95% CI)	NPV% (95% CI)	SE % (95% CI)	SP% (95% CI)	PPV% (95% CI)	NPV% (95% CI)
**Female**	64.5 (63.3, 65.7)	84.1 (82.4, 85.8)	93.2 (92.4, 93.9)	41.2 (39.6, 42.8)	40.8 (39.5, 42.0)	95.6 (94.7, 96.6)	96.9 (96.3, 97.6)	32.4 (31.1, 33.6)
**Male**	38.3 (35.7, 40.9)	96.1 (94.6, 97.6)	95.1 (93.3, 97.0)	43.7 (41.2, 46.3)	18.6 (16.5, 20.7)	99.2 (98.6, 99.9)	98.0 (96.3, 99.7)	37.9 (35.6, 40.1)
**Urban**	60.1 (59.0, 61.3)	86.9 (85.5, 88.3)	93.4 (92.6, 94.1)	41.6 (40.2, 43.0)	37.1 (36.0, 38.2)	96.4 (95.7, 97.2)	96.9 (96.3, 97.6)	33.4 (32.2, 34.5)
**Rural**	54.1 (49.0, 59.2)	92.6 (88.6, 96.7)	94.3 (91.2, 97.4)	47.2 (41.7, 52.7)	31.8 (27.0, 36.6)	99.4 (98.2, 100)	99.2 (97.5, 100)	39.2 (34.5, 43.9)
**All cases**	59.8 (58.7, 60.9)	87.3 (86.0, 88.6)	93.4 (92.7, 94.1)	41.9 (40.6, 43.3)	36.8 (35.7, 37.9)	96.6 (95.9, 97.3)	97.0 (96.4, 97.7)	33.7 (32.6, 34.8)
**Confusion matrix for osteoporosis—Rho score of 7**					**Confusion matrix for osteoporosis—Rho score of 8**			
	**SE %** **(95% CI)**	**SP%** **(95% CI)**	**PPV% (95% CI)**	**NPV% (95% CI)**	**SE %** **(95% CI)**	**SP%** **(95% CI)**	**PPV% (95% CI)**	**NPV% (95% CI)**
**Female**	87.2 (85.8, 88.7)	58.3 (57.1, 59.6)	42.0 (40.5, 43.5)	93.0 (92.1, 93.8)	68.9 (66.9, 70.9)	80.2 (79.1, 81.2)	54.5 (52.6, 56.5)	88.2 (87.3, 89.0)
**Male**	63.6 (59.2, 68.0)	84.5 (82.7, 86.3)	55.7 (51.5, 59.9)	88.3 (86.7, 90.0)	38.1 (33.7, 42.5)	95.2 (94.1, 96.3)	70.9 (65.3, 76.5)	83.4 (81.6, 85.1)
**Urban**	82.8 (81.3, 84.4)	63.1 (61.9, 64.2)	43.1 (41.6, 44.5)	91.6 (90.8, 92.4)	63.3 (61.3, 65.2)	82.8 (81.9, 83.7)	55.4 (53.5, 57.3)	87.0 (86.2, 87.8)
**Rural**	82.2 (75.8, 88.7)	74.8 (70.5, 79.0)	52.6 (45.9, 59.3)	92.5 (89.6, 95.4)	60.7 (52.5, 69.0)	90.9 (88.1, 93.7)	69.5 (61.2, 77.8)	87.2 (83.9, 90.4)
**All cases**	82.8 (81.3, 84.3)	63.7 (62.6, 64.8)	43.5 (42.1, 44.9)	91.6 (90.9, 92.4)	63.1 (61.2, 65.0)	83.2 (82.4, 84.1)	56.0 (54.2, 57.8)	87.0 (86.2, 87.8)

Abbreviations: NPV, negative predictive value; PPV, positive predictive value; SE, sensitivity; SP, specificity.

## Discussion

This study provided external validation of the predictive performance of Rho software as an opportunistic screening tool for low BMD, on a large retrospective dataset. The AUCs for predicting low BMD and osteoporosis using the Rho score were 84.0% and 81.5%, respectively. Rho has the ability to analyze routine radiographs and to detect low BMD and osteoporosis in a non-invasive and efficient manner. The tool is highly applicable in large-scale screening programs, where its implementation can prioritize patients for more comprehensive assessments, thereby reducing healthcare costs and improving patient outcomes. The findings support the use of this AI-based software as a reliable clinical tool for opportunistic screening and its broader implementation within rural communities.

The findings of our study align with the initial study that first published the cutoff values and performance of Rho on 3 datasets. In a study by Bilbily et al., AUC of 0.82, 0.87, and 0.89 were reported for the independent datasets.^10^ That study was the first to implement machine learning to identify patients at risk for low BMD using a variety of standard radiographs. However, to date no independent validation, completed by a research group with no overlapping members or financial ties has been performed. The consistency of performance between the initial study and the current independent validation indicates that the results obtained are reproduceable and support the use of Rho as a clinically useful tool for early prediction of low BMD and osteoporosis.

Other studies have applied AI-based algorithms for the detection of osteoporosis using CT scans reporting AUCs ranging from 0.582 to 0.994.^11^ The limitation of these approaches is the implementation on CT modality, which is not easily accessible, especially in rural communities and usually involves higher radiation doses in routine practice than a single plain radiograph.^10^ Further, many more plain films are performed than CT in daily practice, limiting the use of CT as an opportunistic modality. The use of both modalities could be useful in broadening the number of eligible individuals screened for osteoporosis.

Performance stratified by radiograph location demonstrated highest performance for predicting low BMD with pelvic, thoracic, and lumbar radiographs. The AUC for prediction of osteoporosis was higher for pelvic radiographs compared to all other locations (chest, hand, knee, lumbar, and thoracic). It is possible that the higher AUC of the pelvic region may be due in part to it being a critical anatomical site for bone density evaluation. Several studies have demonstrated higher prevalence of osteoporosis using local DXA scanning at the pelvic and hip site.^17^^,^^18^ Furthermore, BMD was found to be lower in the pelvic site compared to other anatomical regions.^19^ While the performance of Rho on the pelvic radiograph demonstrated greater AUC, the other anatomical locations still demonstrated high prediction accuracies maintaining their utility as opportunistic screening locations.

The AUC for the prediction of low BMD and osteoporosis in both males and females indicate a strong predictive accuracy. However, for the optimal Rho score for prediction of low BMD and osteoporosis differed between males and females, a factor that clinicians should be aware of when interpreting Rho scores. The greater Rho score cutoffs for females may be attributed to various factors including different physiological bone structure and composition.^20^^,^^21^ The differences in Rho score cutoffs for both low BMD and osteoporosis screening between males and females sheds light on the importance of considering sex-differences in the implementation of AI tools.^22^^,^^23^ It is crucial to adjust for these disparities in clinical practice to ensure optimal results and prevent sex-based biases.

The performance of Rho in the rural and urban subgroup yielded intriguing findings. The distribution of the populations among the different Rho score categories was similar across both groups. The AUC values in low BMD and osteoporosis screening in the rural population further demonstrated the clinical utility of Rho. ROC differences were statically significant between rural and urban groups, but overall, clinical performance was consistently good in both groups (AUC > 0.8). This indicates that the software is widely applicable, demonstrating accuracy in both urban and rural populations. Although, prospective patients still need to get DXA, positive Rho screening would greatly enhance referral accuracy.

The high selection accuracy of Rho based on opportunistic screening of plain films has important clinical implications. Rho can serve as a reliable tool for early prediction of low BMD and osteoporosis facilitating earlier diagnosis and treatment thereby reducing the incidence of fractures and other complications.^2^ Our data also shows that Rho can be reliably utilized in rural communities, who traditionally have a greater prevalence of osteoporosis and lower screening rates^6^^,^^7^ due to reduced DXA modality access. The software’s reliably high performance among the different population groups supports the notion that AI tools, such as Rho, could enhance accessibility to healthcare and bridge the gap in osteoporosis detection and management in these communities. The Rho score provides a reliable indication to clinicians to conduct additional fracture risk assessment which may include future DXA scans. The manufacturer suggests a threshold of 6 for prediction of low BMD, which we confirmed, however our sex specific analysis suggested that a lower threshold of 5 may be warranted in male patients. Selection of alternative Rho thresholds based on a particular patient population or DXA capacity allows clinicians to determine their own site-specific trade-off between sensitivity and specificity. Rho analysis is a valuable opportunistic screening tool for individuals over 50 yr of age and may be particularly useful for those in the 50-65 age group who would have limited access to DXA testing unless additional risk factors are present.

Although the use of a single-center study for external validation may be considered a potential limitation to the generalizability of our findings, the included dataset encompasses a broad demographic range from both urban and rural settings, thereby enhancing the generalizability of the results. Importantly, we did not collect information on manufacturer or model of X-ray systems and technical parameters. DXA values can vary due to manufacturers, operators, and patient factors, but it’s still the gold standard in BMD assessment. Previous validation of Rho across systems and parameters has been published.^10^ Additionally, definitions of low BMD and osteoporosis are not dependent on manufacturer, and a clinician likely does not interpret a T-score based on manufacturer. In future studies, a larger, multi-center cohort that encompasses a greater proportion of rural patients can allow for greater insights on how socioeconomic differences affect the utility of an AI-based screening tool. Another potential limitation is that we did not perform a cost-benefit analysis of implementing Rho in routine practice. Given the retrospective nature of our assessment participant selection was biased to those for whom assessment of BMD was assumed to be relevant, as they had a clinical indication for DXA. This resulted in an overrepresentation of participants with low BMD compared to the normal population. However, we found that Rho demonstrated strong screening performance in those with normal BMD as well. Finally, DXA is an imperfect reference standard due to its moderate association with fracture risk. Future longitudinal studies will be required to determine the utility of Rho in predicting fracture outcomes. The retrospective nature of the study does not allow for evaluation of long-term outcomes of using Rho compared to the existing standard of care. Rho primarily focuses on the evaluation of low BMD and does not have the ability to detect fractures. Although by screening individuals at high risk of low BMD, the tool helps to identify patients who may be at a higher risk for fractures. Additional prospective studies including measures, such as fracture rates, and osteoporosis treatment or referral to specialists would be of value to explore the AI tool’s potential to guide further risk assessment of osteoporosis.

### Conclusion

The evaluation of Rho in this cohort indicates its accuracy as an opportunistic screening tool for low BMD and osteoporosis. The software demonstrates strong screening performance with consistent findings among radiograph locations and demographics and will be particularly useful in communities with inadequate access to DXA scans. Integration of AI tools like Rho into standard routine practice can increase screening at a broader level, reducing the disparities in care and ultimately improving patient outcomes and healthcare burden of osteoporosis.

## Supplementary Material

JBMR_Supplamentary_Table_1_ziaf191

## Data Availability

Data will be made available on request following ethics compliance.
