# Supplementary material for: Evaluating an Artificial Intelligence software for opportunistic low bone mineral density and osteoporosis screening: a validation study
Source: JBMR Plus. 2025 Dec 15;10(2):ziaf191. doi: 10.1093/jbmrpl/ziaf191 (PMC12790272; doi:10.1093/jbmrpl/ziaf191)
Supplement: JBMR_Supplamentary_Table_1_ziaf191 [file jbmr_supplamentary_table_1_ziaf191.docx]

Supplementary Table 1: Confusion matrix for a Rho score of 7 or 8.

| **Confusion Matrix for Low BMD - Rho Score of 7** | | | | | **Confusion Matrix for Low BMD - Rho Score of 8** | | | | |
| --- | --- | --- | --- | --- | --- | --- | --- | --- | --- |
|  | **True Positive** | **False Negative** | **False Positive** | **True Negative** |  | **True Positive** | **False Negative** | **False Positive** | **True Negative** |
| Female | 3948 | 2176 | 289 | 1525 | Female | 2496 | 3628 | 79 | 1735 |
| Male | 507 | 818 | 26 | 636 | Male | 246 | 1079 | 5 | 657 |
| Urban | 4256 | 2825 | 303 | 2010 | Urban | 2625 | 4456 | 83 | 2230 |
| Rural | 199 | 169 | 12 | 151 | Rural | 117 | 251 | 1 | 162 |
| All cases | 4455 | 2994 | 315 | 2161 | All cases | 2742 | 4707 | 84 | 2392 |
| **Confusion Matrix for Osteoporosis - Rho Score of 7** | | | | | **Confusion Matrix for Osteoporosis - Rho Score of 8** | | | | |
|  | **True Positive** | **False Negative** | **False Positive** | **True Negative** |  | **True Positive** | **False Negative** | **False Positive** | **True Negative** |
| Female | 1778 | 261 | 2459 | 3440 | Female | 1404 | 635 | 1171 | 4728 |
| Male | 297 | 170 | 236 | 1284 | Male | 178 | 289 | 73 | 1447 |
| Urban | 1964 | 407 | 2595 | 4428 | Urban | 1500 | 871 | 1208 | 5815 |
| Rural | 111 | 24 | 100 | 296 | Rural | 82 | 53 | 36 | 360 |
| All cases | 2075 | 431 | 2695 | 4724 | All cases | 1582 | 924 | 1244 | 6175 |
